# Supplementary material for: Improving continence management for people with dementia in the community in Aotearoa, New Zealand: Protocol for a mixed methods study
Source: PLoS One. 2023 Jul 18;18(7):e0288613. doi: 10.1371/journal.pone.0288613 (PMC10353819; doi:10.1371/journal.pone.0288613)
Supplement: S1 Appendix — (DOCX) [file pone.0288613.s001.docx]

**S1 APPENDIX**

**FOCUS GROUP QUESTIONS**

1. Vignette: *Molly has dementia and lives at home with her spouse, Harry. Molly uses pull-up continence pants, but despite this, Harry finds he needs to wake Molly every couple of hours in order to take her to the toilet to avoid a wet bed in the morning.*What solutions would be offered to promote continence or manage incontinence for Molly and to support Harry living at home? (10 minutes)
2. Vignette: *Ann lives alone and has recently developed memory problems. Ann uses a walking stick. She has a venous leg ulcer and has had a nurse visiting to changing her dressing. Over the last few weeks, it has become increasingly noticeable to the nurse that there is a smell of urine about Ann and in her home. On her last visit the nurse notices that Ann is sitting on some newspaper.* What solutions would be offered to promote continence or manage incontinence for Ann living at home? (10 minutes)
3. Vignette: *Mr Wilson has moderate dementia and lives at home with his wife. During one of his visits to the GP his wife mentions that he has recently started to urinate in unusual places around the home, rather than using the toilet. He is very resistant to accept help from his wife.* What solutions would be offered to promote continence or manage incontinence for Mr Wilson living at home, and to support his wife? (10 minutes)
4. Vignette: *Mrs Kahiwa has memory problems and is supported by her whānau. During a kaumātua ora education day held by a local Māori service provider, her daughter describes an issue that she is facing – for the past few months she has been finding wet underwear hidden in drawers, or under the bed.  Yesterday she found faeces wrapped in clothing hidden in a wardrobe.* What solutions would be offered to promote continence or manage incontinence for this kuia and whānau living at home? (10 minutes)
5. From professional and clinical experience, are there strategies/advice/ interventions/aids/support/technology that appear to be more acceptable and effective in promoting continence or managing incontinence for people living with dementia at home? (10 minutes)
6. From professional and clinical experience, are there gaps in the knowledge and/or provision to appropriately support people living with dementia and their carers in promoting continence or managing incontinence? (10 minutes)

Approved by Auckland Health Research Ethics Committee on 20/10/2021 for three years. Reference Number AH23247
