# Supplementary material for: Improving continence management for people with dementia in the community in Aotearoa, New Zealand: Protocol for a mixed methods study
Source: PLoS One. 2023 Jul 18;18(7):e0288613. doi: 10.1371/journal.pone.0288613 (PMC10353819; doi:10.1371/journal.pone.0288613)
Supplement: S2 Appendix — (DOCX) [file pone.0288613.s002.docx]

**S2 APPENDIX: TOPIC GUIDE PLWD CROSS-SECTIONAL INTERVIEW**

**Note to interviewers –** all questions are **about the PLWD**. Those that can only be answered by the PLWD (i.e. those that are subjective) are marked with *. Remember there is an option to split interview between two visits if needed.

*To PLWD –*

1. Can you tell me a little bit about yourself? (*whatever the participant would like to tell you)*

***DEMOGRAPHIC SURVEY ITEMS ASKED HERE***

1. How are you managing to do all the things that you used to do, or would like to do around the home at the present time?

*Conversational probes (this is to get a general idea about how the PLWD manages, not a huge amount of detail is required)*

- *Activities of daily living such as eating and drinking, dental care, washing, bathing, dressing, physical activity and exercising, personal grooming, home maintenance, laundry, other household chores.*
- *Aids, adaptations or technology used to support activities of daily living*

1. How are you managing to do the things you would like to do in the community at the present time? *(this is to get a general idea about how the PLWD manages, not a huge amount of detail is required)*

*Conversational probes*

- *Physical activity and exercise outside of the home*
- *Telling people about memory problems*
- *Eating and drinking out*
- *Maintaining relationships with people in the community*
- *Participation in religious activity/church/spiritual groups*
- *Travelling and transport*
- *Aids or strategies that are used to support these things*

4.* We’ve been talking about how you manage to do things at home and in the community, I was wondering, what are your sources of hope, strength, comfort and peace? For example, a religious or spiritual beliefs acts as a source of comfort and strength for some people in dealing with life's ups and downs.

*Conversational probes*

- *Part of an* ***organized religion*** *if so what is it?*
- ***Personal*** *spirituality/ practices independent of organized religion*
- *How important is spirituality, faith or religion is to the person*

5. Today, I’m going to be talking a lot about getting to and using the toilet, the accidental loss or leaking of urine (e.g. peeing pants, wetting the bed) the accidental loss or leaking faeces, and constipation. Do you have any preferences about how I should refer to these? ***Note to interviewer: From this point on use the terminology preferred by the participant.***

***M-ACE QUESTIONS ASKED HERE***

**SECTION: Toileting**

1. Some people sometimes experience problems with going to the toilet (e.g. finding the toilet in time, managing clothing) do you ever have those sort of problems?

*Conversational probes for types of problems (to be used if participant isn’t forthcoming with describing issues. Not all of these have to be covered with each person e.g. last bullet points only likely at later stages of dementia).*

- *Can’t find the toilet*
- *Finds the toilet but can’t use it*
- *Hides evidence of accidents*
- *Wont’ sit on the toilet*
- *Uses inappropriate receptacle/place*
- *Can’t manage clothing*
- *Faecal smearing*
- *Not recognizing the urge or desire to act on it*

*Conversational probe about frequency (e.g. how often does that happen?)*

***If there are toileting problems ask topics 7-11 if not skip to 12***

7.* Do these problems with toileting interfere with your everyday life (PLWD)?

*Conversational probes*

- *Whether using the toilet is always on their mind*
- *Whether they feel embarrassed*
- *Whether they try to make sure they know where the toilets are (e.g. in the community or when visiting others)*
- *Whether they make plans taking toileting problems into account*
- *Whether toileting problems cause them to stay home more often than they would like*

1. What helps you to deal with that? How do you manage?

*Conversational probes*

- *Types of strategies for addressing these problems*
- *Are there different strategies adopted by PLWD and supporter*

1. Are the strategies you use useful?

*Conversational probes*

- *Preferences about different strategies do they differ between PLWD and supporter*
- *Feelings about different strategies e.g. How do you feel about managing in that way?*
- *Perceived effectiveness and whether this differs between PLWD and supporter.*

1. Some people discuss these sorts of problems with their family doctor or with a district or continence nurse. Have you sought advice or help on this from any professional?

*Conversational probes:*

- *With whom (GP community nurse, occupational therapist, physio, mental health nurse)*
- *What support/information was provided*
- *Was this enough support/information: perceptions of efficacy, usefulness, nuisance from the perspectives of PLWD and supporter, any unmet needs*
- *Views on experience of discussing or receiving help from health professionals*
- *Any examples of ‘normalisation’ of UI by health professionals*

11a. * Overall, if you had to summarise the impact that these toileting issues have on your life, how would you rate this? Please could you point to the one of the faces on the card that best reflects the impact that toileting issues have on your life, where the green face represent no impact, the yellow face represents some impact and the red face, a great deal of impact (PLWD).

**SHOWCARD A**

11b. **ONLY ASK IF THE SUPPORTER IS PRESENT AND HAS NOT REQUESTED A SEPARATE INTERVIEW:** Overall, if you had to summarise the impact that these toileting issues have on your life, how would you rate this? Please could you point to the one of the faces on the card that best reflects the impact that toileting issues have on your life, where the green face represent no impact, the yellow face represents some impact and the red face, a great deal of impact (supporter).

**SHOWCARD A**

**SECTION: Nocturia**

1. Bladder control, urinary incontinence or having to get up at night to urinate during the night can be a problem for some people. Thinking about how you have been recently, how often do you pass urine during the night from going to bed to sleep until you get up in the morning?

13. * Having to get up at night to urinate can impact on other areas of your life. I wonder if you could describe whether having to get up at night to urinate has had any impact on you recently (PLWD)?

*Conversational Probes*

- *Made it difficult to concentrate the next day*
- *Feel generally low in energy*
- *Difficult to sleep at night, disturbed sleep or getting back to sleep, required to nap the next day*
- *Less productive the next day*
- *Participate less in the activities you enjoy*
- *Careful about how much you drink or when you drink*
- *Worried about disturbing other in the house (e.g. their sleep)*
- *Falling at night*
- *Ask how often each happens (e.g. frequently, occasionally)*

14a. * Overall, if you had to summarise the impact that nocturia had on your life, how would you rate this? Please could you point to the one of the faces on the card that best reflects the impact that toileting issues have on your life, where the green face represent no impact, the yellow face represents some impact and the red face, a great deal of impact (PLWD).

**SHOWCARD A**.

14b. **ONLY ASK IF THE SUPPORTER IS PRESENT AND HAS NOT REQUESTED A SEPARATE INTERVIEW:** Overall, if you had to summarise the impact that nocturia had on your life, how would you rate this? Please could you point to the one of the faces on the card that best reflects the impact that toileting issues have on your life, where the green face represent no impact, the yellow face represents some impact and the red face, a great deal of impact (supporter).

**SHOWCARD A**.

**SECTION: UI**

1. Urinary incontinence means a person leaks urine by accident (pees their pants or wets the bed: use participant’s terminology). Do you ever leak urine?

***Note: If participant experiences UI continue with topics below.***

***If participant has experienced nocturia that disturbs sleep skip to 19.***

***If neither nocturia or UI skip to 23.***

1. When does this urine leak?

*Conversation probes*

- *Before can get to toilet*
- *When cough or sneeze*
- *When asleep/at night/bed wetting*
- *Physically active/exercising*
- *Finished dressing and are dressed*
- *No obvious reasons*

17*. Do problems with leaking urine interfere with your everyday life (PLWD)?

*Conversational probes*

- *Whether using the toilet is always on their mind*
- *Whether they feel embarrassed*
- *Whether they try to make sure they know where the toilets are*
- *Whether they make plans taking UI into account*
- *Whether UI cause them to stay home more often than they would like*

18a.* Overall, if you had to summarise the impact that leaking urine has on your life, how would you rate this? Please could you point to the one of the faces on the card that best reflects the impact that leaking urine has on your life, where the green face represents no impact, the yellow face represents some impact and the red face, a great deal of impact (PLWD).

**SHOWCARD A**

18b. **ONLY ASK IF THE SUPPORTER IS PRESENT AND HAS NOT REQUESTED A SEPARATE INTERVIEW:** Overall, if you had to summarise the impact that leaking urine has on your life, how would you rate this? Please could you point to the one of the faces on the card that best reflects the impact that leaking urine has on your life, where the green face represents no impact, the yellow face represents some impact and the red face, a great deal of impact (supporter).

**SHOWCARD A**

**SECTION: Strategies for UI & Nocturia**

***Note: if person experiences both UI and nocturia, probe about strategies for both in topic areas 19-22.***

1. What helps you to deal with [UI and/or nocturia]? How do you manage?

*Conversational probes for*

- *Types of strategies for addressing these problems e.g. incontinence pads, pull-ups, male continence sheath, absorbent bed pad, waterproof mattress protector, catheter, timing of drinking fluids/restricting fluid intake,*
- *For continence products: whether they are provided free or whether they have to purchase them. If they are free, do they have to top up with purchases explore whether provision is inadequate or inappropriate (see below)*
- *Toileting programmes, pelvic floor exercises, bladder retraining*
- *Are there different strategies adopted by PLWD and supporter*
- *Ability to clean up after an episode of urinary incontinence or leakage*

1. Are the strategies you use for [UI and/or nocturia] useful?

*Conversational probes*

- *Preferences about different strategies do they differ between PLWD and supporter*
- *Feelings about different strategies e.g. How do you feel about managing in that way?*
- *Perceived effectiveness and whether this differs between PLWD and supporter.*
- *Are the PLWD and supporter able to use these strategies when they leave the home e.g. visiting friends, shopping, eating out, leisure activities.*

21. FOR THOSE USING INCONTINENCE AIDS ONLY: Are there any particular challenges that you face when using incontinence products?

*Conversational probes*

- *Appeal/resistance: if continence aids are useful and acceptable to the PLWD and caregiver, whether they would prefer something else (e.g. pull ups instead of pads)*
- *Product performance: Leakage from products (e.g. pad or continence sheets), device movement/loss of adhesion when walking*
- *Accurate, effective application (by self or carers)*
- *Difficult anatomy, thin skin, excessive tissue/fat, creases and skin folds*
- *Options for sustainable or washable/reusable products (e.g. pads or knickers)*
- *Odour*
- *Skin health e.g. friction, pressure sores, dermatitis/rash, removal of device without skin trauma*
- *Reduced or no potential for toileting*
- *Taking spare products and/or disposal when out*
- *Method and cost of disposal at home*

1. Some people discuss these sorts of problems with their family doctor or with a district or continence nurse. Have you sought advice or help on this from any professional?

*Conversational probes:*

- *With whom (GP community nurse, occupational therapist, physio, mental health nurse)*
- *What support/information was provided*
- *Was this enough support/information: perceptions of efficacy, usefulness, nuisance from the perspectives of PLWD and supporter, any unmet needs*
- *Views on experience of discussing or receiving help from health professionals*
- *Any examples of ‘normalisation’ of UI by health professionals*

**SECTION: Bowels**

1. Some people experience problems with their bowels, getting constipated and then sometimes very loose motions, feacal leakage or uncontrollable wind, do you ever have those sorts of problems?

*If yes, prompt what type of problem and SHOWCARD B and ask participant to indicate bowel movements*

***SHOWCARD B***

***Note: If participant experiences FI or constipation continue with topics below, otherwise skip to closing section topic 31-32***

1. Thinking about how you have been recently, how often does this happen?

*Conversational probe for each type of bowel problem mentioned*

*FOR FI Conversation probes around when this happens*

- *Small amounts leaking all the time*
- *When asleep/at night in bed*
- *When passive e.g. Just sitting*
- *Before they can get to the toilet*
- *At particular points (e.g. after meals)*

25.* Do these problems with [leakage of faeces or constipation] interfere with your everyday life (PLWD)?

*Conversational probes*

- *Whether using the toilet is always on their mind*
- *Whether they feel embarrassed*
- *Whether they try to make sure they know where the toilets are*
- *Whether they make plans taking FI/constipation into account*
- *Whether FI/constipation causes them to stay home more often than they would like*

*For night time events, if mentioned:*

- *Made it difficult to concentrate the next day*
- *Feel generally low in energy*
- *Difficult to sleep at night, disturbed sleep or getting back to sleep, required to nap the next day*
- *Less productive the next day*
- *Participate less in the activities you enjoy*
- *Careful about how much you drink or when you drink*
- *Concern about disturbing other in the house (e.g. their sleep)*

1. What helps you to deal with [constipation or faecal incontinence]? How do you manage?

***If both then prompt around types of strategies used for each in topic areas 26-29***

*Conversational probes*

- *Types of strategies for addressing these problems e.g. pads, incontinence sheets, colostomy.*
- *For continence products: whether they are provided free or whether they have to purchase them*
- *Are there different strategies adopted by PLWD and supporter*
- *Ability to clean up after an episode of faecal incontinence or leakage.*

1. Are the strategies you use useful?

*Conversational probes*

- *Preferences about different strategies do they differ between PLWD and supporter*
- *Feelings about different strategies e.g. How do you feel about managing in that way?*
- *Perceived effectiveness and whether this differs between PLWD and supporter.*
- *Are the PLWD and supporter able to use these strategies when they leave the home e.g. visiting friends, shopping, eating out, leisure activities.*

28. FOR THOSE USING INCONTINENCE AIDS ONLY: Are there any particular challenges that you face when using incontinence products for faecal incontinence?

***Note: If these topics have been covered in relation to UI, only ask if there are any particular challenges associated with FI that are different.***

*Conversational probes*

- *Appeal/resistance: if continence aids are useful and acceptable to the PLWD and caregiver, whether they would prefer something else (e.g. pull ups instead of pads)*
- *Product performance: Leakage from products, device movement/loss of adhesion when walking*
- *Accurate, effective application (by self or carers)*
- *Difficult anatomy, thin skin, excessive tissue/fat, creases and skin folds*
- *Options for sustainable or washable/reusable products (e.g. pads or knickers)*
- *Odour*
- *Skin health e.g. friction, pressure sores, dermatitis/rash, removal of device without skin trauma*
- *Reduced or no potential for toileting*
- *Taking spare products and/or disposal when out*
- *Method and cost of disposal at home*

1. Some people discuss these sorts of problems with their family doctor or with a district or continence nurse. Have you sought advice or help on this from any professional?

*Conversational probes:*

- *With whom (GP community nurse, occupational therapist, physio, mental health nurse)*
- *What support/information was provided*
- *Was this enough support/information: perceptions of efficacy, usefulness, nuisance from the perspectives of PLWD and supporter, any unmet needs*
- *Views on experience of discussing or receiving help from health professionals*
- *Any examples of ‘normalisation’ of UI by health professionals*

30a.* Overall, if you had to summarise the impact that [faecal incontinence, constipation] has on your life, how would you rate this? Please could you point to the one of the faces on the card that best reflects the impact that [faecal incontinence, constipation] has on your life, where the green face represents no impact, the yellow face represents some impact and the red face, a great deal of impact (PLWD).

**SHOWCARD A**

***Note, ask this twice if person experiences both faecal incontinence and constipation, once for each.***

30b. **ONLY ASK IF THE SUPPORTER IS PRESENT AND HAS NOT REQUESTED A SEPARATE INTERVIEW:** Overall, if you had to summarise the impact that [faecal incontinence, constipation] has on your life, how would you rate this? Please could you point to the one of the faces on the card that best reflects the impact that [faecal incontinence, constipation] has on your life, where the green face represents no impact, the yellow face represents some impact and the red face, a great deal of impact (PLWD).

**SHOWCARD A**

***Note, ask this twice if PLWD experiences both faecal incontinence and constipation, once for each.***

**SECTION: Spirituality, QOL & Close**

1. Is there anything you would like to tell me about your spirituality, faith or religion that helps or hinders you in dealing with your memory problems and continence issues?

*Conversational probes*

- *Whether they find comfort in religion or spiritual beliefs*
- *Whether illness has strengthened religion or spiritual beliefs.*

32a.* We’ve talked about lots of things today. Thinking about all of these things in the last week, how would you rate your quality of life overall? Please could you point to the one of the faces on the card that best reflects how you feel, where the faces represent good, fair and poor quality of life.

**SHOWCARD C**

32b.  **ONLY ASK IF THE SUPPORTER IS PRESENT AND HAS NOT REQUESTED A SEPARATE INTERVIEW:** We’ve talked about lots of things today. Thinking about all of these things in the last week, how would you rate your quality of life overall? Please could you point to the one of the faces on the card that best reflects how you feel, where the faces represent good, fair and poor quality of life.

**SHOWCARD C**

Approved by Southern Health and Disability Ethics Committee on 28/04/2022 for three years. Reference Number 11658
