# Supplementary material for: Improving continence management for people with dementia in the community in Aotearoa, New Zealand: Protocol for a mixed methods study
Source: PLoS One. 2023 Jul 18;18(7):e0288613. doi: 10.1371/journal.pone.0288613 (PMC10353819; doi:10.1371/journal.pone.0288613)
Supplement: S3 Appendix — (DOCX) [file pone.0288613.s003.docx]

**S3 APPENDIX: TOPIC GUIDE CAREGIVER CROSS-SECTIONAL INTERVIEW**

**(SUPPORTERS WHO HAVE ALREADY BEEN INTERVIEWED WITH PLWD)**

We’ve talked to the person you provide support to (and you [if present]) about issues around toileting, urinary and faecal incontinence. We wanted also to provide an opportunity for you to discuss anything that you wished to raise with us, that you felt unable to discuss in front of [name]. I’d also like to know how these issues impact on your life.

**Note to interviewer: It is only necessary to ask question relating to the areas which were already discussed as problematic (e.g. if faecal incontinence was not an issue there is no need to ask all of the questions relating to bowels). However, it is probably worth checking the first question of each section in case the PLWD present did not reveal an issue that the supporter does want to raise (e.g. if PLWD said no issues with bowels, but supporter does think there is an issue with bowels).**

**SECTION: TOILETTING**

1. Is there anything extra that you wish to talk about with going to the toilet (e.g. finding the toilet in time, managing clothing) that you have not already told us about?

*Conversational probes for types of problems (to be used if participant isn’t forthcoming with describing issues. Not all of these have to be covered with each person e.g. last bullet points only likely at later stages of dementia).*

- *Can’t find the toilet*
- *Finds the toilet but can’t use it*
- *Hides evidence of accidents*
- *Wont’ sit on the toilet*
- *Uses inappropriate receptacle/place*
- *Can’t manage clothing*
- *Faecal smearing*
- *Not recognizing the urge or desire to act on it*

*Conversational probe about frequency (e.g. how often does that happen?) t*oileting?

2. Do these problems with toileting interfere with your everyday life (supporter)?

*Conversational probes*

- *Whether they feel embarrassed going out with PLWD or dealing with intimate tasks*
- *Whether they try to make sure they know where the toilets are (e.g. in the community or when visiting others)*
- *Whether they make plans taking toileting problems into account*
- *Whether toileting problems cause them to stay home more often than they would like*

1. Are there any strategies that help you deal with that (that you have not already mentioned) that you would like to tell me about? How do you manage?

*Conversational probes*

- *Types of strategies for addressing these problems*

1. **ONLY ASK IF NEW STRATEGIES ARE RAISED:** Are the strategies you use useful?

*Conversational probes*

- *Feelings about different strategies e.g. How do you feel about managing in that way?*

1. Some people discuss these sorts of problems with their family doctor or with a district or continence nurse. Is there anything you want to tell me about (that you haven’t already raised) about seeking advice or help on this from any professional?

*Conversational probes:*

- *With whom (GP community nurse, occupational therapist, physio, mental health nurse)*
- *What support/information was provided*
- *Was this enough support/information: perceptions of efficacy, usefulness, nuisance from the perspectives of supporter, any unmet needs*
- *Views on experience of discussing or receiving help from health professionals*
- *Any examples of ‘normalisation’ of UI by health professionals*

6. **ONLY ASK IF THE SUPPORTER HAS NOT ALREADY BEEN ASKED IN A JOINT INTERVIEW:** Overall, if you had to summarise the impact that these toileting issues have on **your lif**e, how would you rate this? Please could you point to the one of the faces on the card that best reflects the impact that toileting issues have on your life, where the green face represent no impact, the yellow face represents some impact and the red face, a great deal of impact (supporter not PLWD).

**SHOWCARD A**

**SECTION: Nocturia**

1. We’ve talked about bladder control, urinary incontinence or having to get up at night to urinate during the night with [name]. Is there anything you would like to say about [name] having to get up at night to urinate and how this impacts on **your** life? (supporter)

*Conversational Probes*

- *Made it difficult to concentrate the next day*
- *Feel generally low in energy*
- *Difficult to sleep at night, disturbed sleep or getting back to sleep, required to nap the next day*
- *Less productive the next day*
- *Participate less in the activities you enjoy*

1. **ONLY ASK IF THE SUPPORTER HAS NOT ALREADY BEEN ASKED IN A JOINT INTERVIEW:** Overall, if you had to summarise the impact that nocturia had on **your** life, how would you rate this? Please could you point to the one of the faces on the card that best reflects the impact that toileting issues have on your life, where the green face represent no impact, the yellow face represents some impact and the red face, a great deal of impact (supporter not PLWD).

**SHOWCARD A**.

**SECTION: UI**

1. Urinary incontinence means a person leaks urine by accident (pees their pants or wets the bed/ use participant’s terminology). Is there anything extra you want to tell me about [name of PLWD] and urinary incontinence?
2. Do problems with [name of PLWD] leaking urine interfere with your everyday life (supporter)?

*Conversational probes*

- *Whether they feel embarrassed taking PLWD out or dealing with intimate tasks*
- *Whether they try to make sure they know where the toilets are when they take [name] out*
- *Whether they make plans taking UI into account*
- *Whether UI cause them to stay home more often than they would like*
- *Extra laundry, house cleaning, financial outlay*

1. **ONLY ASK IF THE SUPPORTER HAS NOT ALREADY BEEN ASKED IN A JOINT INTERVIEW:** Overall, if you had to summarise the impact that [name of PLWD] leaking urine has on your life, how would you rate this? Please could you point to the one of the faces on the card that best reflects the impact that leaking urine has on your life, where the green face represents no impact, the yellow face represents some impact and the red face, a great deal of impact (supporter).

**SHOWCARD A**

**SECTION: Strategies for UI & Nocturia**

***Note: if person living with dementia experiences both UI and nocturia, probe about strategies for both in topic areas 12-15.***

1. Is there anything extra that you would like to tell me about what helps you to deal with [UI and/or nocturia]? How do you manage?

*Conversational probes for*

- *Types of strategies for addressing these problems e.g. incontinence pads, pull-ups, male continence sheath, absorbent bed pad, waterproof mattress protector, catheter, timing of drinking fluids/restricting fluid intake,*
- *For continence products: whether they are provided free or whether they have to purchase them. If they are free, do they have to top up with purchases explore whether provision is inadequate or inappropriate (see below)*
- *Toileting programmes, pelvic floor exercises, bladder retraining*
- *Are there different strategies adopted by PLWD and supporter*
- *Ability to clean up after an episode of urinary incontinence or leakage*

1. **ONLY ASK IF NEW STRATEGIES ARE RAISED:** Are the strategies you use for [UI and/or nocturia] useful?

*Conversational probes*

- *Preferences about different strategies*
- *Feelings about different strategies e.g. How do you feel about managing in that way?*
- *Perceived effectiveness*
- *Are the PLWD and supporter able to use these strategies when they leave the home e.g. visiting friends, shopping, eating out, leisure activities.*

1. **FOR THOSE USING INCONTINENCE AIDS ONLY:** Are there any particular challenges that you face when using incontinence products that you have not already mentioned?

*Conversational probes*

- *Appeal/resistance: if continence aids are useful and acceptable, whether they would prefer something else (e.g. pull ups instead of pads)*
- *Product performance: Leakage from products (e.g. pad or continence sheets), device movement/loss of adhesion when walking*
- *Accurate, effective application (by self or carers)*
- *Difficult anatomy, thin skin, excessive tissue/fat, creases and skin folds*
- *Options for sustainable or washable/reusable products (e.g. pads or knickers)*
- *Odour*
- *Skin health e.g. friction, pressure sores, dermatitis/rash, removal of device without skin trauma*
- *Reduced or no potential for toileting*
- *Taking spare products and/or disposal when out*
- *Method and cost of disposal at home*

1. Some people discuss these sorts of problems with their family doctor or with a district or continence nurse. Is there anything you want to tell me about (that you haven’t already raised) about seeking advice or help on this from any professional? *Conversational probes:*

- *With whom (GP community nurse, occupational therapist, physio, mental health nurse)*
- *What support/information was provided*
- *Was this enough support/information: perceptions of efficacy, usefulness, nuisance from the perspectives of PLWD and supporter, any unmet needs*
- *Views on experience of discussing or receiving help from health professionals*
- *Any examples of ‘normalisation’ of UI by health professionals*

**SECTION: Bowels**

1. Is there anything extra that you would like to tell me about what helps you to deal with [name] problems with their bowels/constipation/loose motions/feacal leakage or uncontrollable wind?

*If yes, prompt what type of problem and SHOWCARD B and ask participant to indicate bowel movements*

***SHOWCARD B***

***Note: If PLWD experiences FI or constipation continue with topics below, otherwise skip to closing section topic 24***

1. Is there anything extra that you would like to tell me about when this happens/how frequently this happens?

*Conversational probe for each type of bowel problem mentioned*

*FOR FI Conversation probes around when this happens*

- *Small amounts leaking all the time*
- *When asleep/at night in bed*
- *When passive e.g. Just sitting*
- *Before they can get to the toilet*
- *At particular points (e.g. after meals)*

1. Do these problems with [leakage of faeces or constipation] interfere with your everyday life (supporter)?

*Conversational probes*

- *Whether they feel embarrassed taking PLWD out or dealing with intimate tasks*
- *Whether they try to make sure they know where the toilets are*
- *Whether they make plans taking FI/constipation into account*
- *Whether FI/constipation causes them to stay home more often than they would like*

*For night time events, if mentioned:*

- *Made it difficult to concentrate the next day*
- *Feel generally low in energy*
- *Difficult to sleep at night, disturbed sleep or getting back to sleep, required to nap the next day*
- *Less productive the next day*
- *Participate less in the activities you enjoy*
- *Careful about how much you drink or when you drink*
- *Concern about disturbing other in the house (e.g. their sleep)*

1. Is there anything extra you’d like to tell me about what helps you to deal with [constipation or faecal incontinence]? How do you manage?

***If both then prompt around types of strategies used for each in topic areas 20-22***

*Conversational probes*

- *Types of strategies for addressing these problems e.g. pads, incontinence sheets, colostomy.*
- *For continence products: whether they are provided free or whether they have to purchase them*
- *Are there different strategies adopted by PLWD and supporter*
- *Ability to clean up after an episode of faecal incontinence or leakage.*

1. **ONLY ASK IF NEW STRATEGIES ARE RAISED:** Are the strategies you use useful?

*Conversational probes*

- *Preferences about different strategies do they differ between PLWD and supporter*
- *Feelings about different strategies e.g. How do you feel about managing in that way?*
- *Perceived effectiveness and whether this differs between PLWD and supporter.*
- *Are the PLWD and supporter able to use these strategies when they leave the home e.g. visiting friends, shopping, eating out, leisure activities.*

1. **FOR THOSE USING INCONTINENCE AIDS ONLY:** Are there any particular challenges that you face when using incontinence products for faecal incontinence that you have not already mentioned?

***Note: If these topics have been covered in relation to UI, only ask if there are any particular challenges associated with FI that are different.***

*Conversational probes*

- *Appeal/resistance: if continence aids are useful and acceptable to the PLWD and caregiver, whether they would prefer something else (e.g. pull ups instead of pads)*
- *Product performance: Leakage from products, device movement/loss of adhesion when walking*
- *Accurate, effective application (by self or carers)*
- *Difficult anatomy, thin skin, excessive tissue/fat, creases and skin folds*
- *Options for sustainable or washable/reusable products (e.g. pads or knickers)*
- *Odour*
- *Skin health e.g. friction, pressure sores, dermatitis/rash, removal of device without skin trauma*
- *Reduced or no potential for toileting*
- *Taking spare products and/or disposal when out*
- *Method and cost of disposal at home*

1. Some people discuss these sorts of problems with their family doctor or with a district or continence nurse. Is there anything you want to tell me about (that you haven’t already raised) about seeking advice or help on this from any professional? *Conversational probes:*

- *With whom (GP community nurse, occupational therapist, physio, mental health nurse)*
- *What support/information was provided*
- *Was this enough support/information: perceptions of efficacy, usefulness, nuisance from the perspectives of PLWD and supporter, any unmet needs*
- *Views on experience of discussing or receiving help from health professionals*
- *Any examples of ‘normalisation’ of UI by health professionals*

1. **ONLY ASK IF THE SUPPORTER HAS NOT ALREADY BEEN ASKED IN A JOINT INTERVIEW:** Overall, if you had to summarise the impact that [faecal incontinence, constipation] has on your life, how would you rate this? Please could you point to the one of the faces on the card that best reflects the impact that [faecal incontinence, constipation] has on your life, where the green face represents no impact, the yellow face represents some impact and the red face, a great deal of impact (PLWD). **SHOWCARD A *Note, ask this twice if PLWD t experiences both faecal incontinence and constipation, once for each.***

**SECTION: Spirituality, QOL & Close**

1. Is there anything you would like to tell me about your spirituality, faith or religion that helps or hinders you in supporting [name] with their memory problems and continence issues?

*Conversational probes*

- *Whether they find comfort in religion or spiritual beliefs*
- *Whether illness has strengthened religion or spiritual beliefs.*

1. **ONLY ASK IF THE SUPPORTER HAS NOT ALREADY BEEN ASKED IN A JOINT INTERVIEW:** We’ve talked about lots of things today. Thinking about all of these things in the last week, how would you rate your quality of life overall? Please could you point to the one of the faces on the card that best reflects how you feel, where the faces represent good, fair and poor quality of life.

**SHOWCARD C**

**(SUPPORTERS WHO HAVE NOT BEEN INTERVIEWED WITH PLWD)**

I’ve talked the person you provide support to about issues around toileting, urinary and faecal incontinence. I also wanted to talk to you to understand your perspective on these issues and how they impact on your life.

**INTRODUCTORY SECTION**

1. Can you tell me a little bit about yourself? (*whatever the participant would like to tell you*
2. How is [name of PLWD] managing to do all the things that they used to do, or would like to do around the home at the present time?

*Conversational probes (this is to get a general idea about how the PLWD manages, not a huge amount of detail is required)*

- *Activities of daily living such as eating and drinking, dental care, washing, bathing, dressing, physical activity and exercising, personal grooming, home maintenance, laundry, other household chores.*
- *Aids, adaptations or technology used to support activities of daily living*

1. How is [name of PLWD] managing to do the things they would like to do in the community at the present time? *(this is to get a general idea about how the PLWD manages, not a huge amount of detail is required)*

*Conversational probes*

- *Physical activity and exercise outside of the home*
- *Telling people about memory problems*
- *Eating and drinking out*
- *Maintaining relationships with people in the community*
- *Participation in religious activity/church/spiritual groups*
- *Travelling and transport*
- *Aids or strategies that are used to support these things*

1. We’ve been talking about how [name of PLWD] manages to do things at home and in the community, I was wondering, what are **your** sources of hope, strength, comfort and peace? For example, a religious or spiritual beliefs acts as a source of comfort and strength for some people in dealing with life's ups and downs.

*Conversational probes*

- *Part of an* ***organized religion*** *if so what is it?*
- ***Personal*** *spirituality/ practices independent of organized religion*
- *How important is spirituality, faith or religion is to the person*

1. Today, I’m going to be talking a lot about getting to and using the toilet, the accidental loss or leaking of urine (e.g. peeing pants, wetting the bed) the accidental loss or leaking faeces, and constipation. Do you have any preferences about how I should refer to these? ***Note to interviewer: From this point on use the terminology preferred by the participant.***

**SECTION: Toileting**

1. Some people sometimes experience problems with going to the toilet (e.g. finding the toilet in time, managing clothing) does [name of PLWD] ever have those sort of problems?

*Conversational probes for types of problems (to be used if participant isn’t forthcoming with describing issues. Not all of these have to be covered with each person e.g. last bullet points only likely at later stages of dementia).*

- *Can’t find the toilet*
- *Finds the toilet but can’t use it*
- *Hides evidence of accidents*
- *Wont’ sit on the toilet*
- *Uses inappropriate receptacle/place*
- *Can’t manage clothing*
- *Faecal smearing*
- *Not recognizing the urge or desire to act on it*
- *Conversational probe about frequency (e.g. how often does that happen?)*

***If there are toileting problems ask topics 7-11 if not skip to 12***

1. Do these problems with toileting interfere with your everyday life (supporter)?

*Conversational probes*

- *Whether they feel embarrassed going out with PLWD or dealing with intimate tasks*
- *Whether they try to make sure they know where the toilets are (e.g. in the community or when visiting others)*
- *Whether they make plans taking toileting problems into account*
- *Whether toileting problems cause them to stay home more often than they would like*

1. What helps you to deal with that? How do you manage?

*Conversational probes*

- *Types of strategies for addressing these problems*
- *Are there different strategies adopted by PLWD and supporter*

1. Are the strategies you use useful?

*Conversational probes*

- *Preferences about different strategies do they differ between PLWD and supporter*
- *Feelings about different strategies e.g. How do you feel about managing in that way?*
- *Perceived effectiveness and whether this differs between PLWD and supporter.*

1. Some people discuss these sorts of problems with their family doctor or with a district or continence nurse. Have you sought advice or help on this from any professional?

*Conversational probes:*

- *With whom (GP community nurse, occupational therapist, physio, mental health nurse)*
- *What support/information was provided*
- *Was this enough support/information: perceptions of efficacy, usefulness, nuisance from the perspectives of PLWD and supporter, any unmet needs*
- *Views on experience of discussing or receiving help from health professionals*
- *Any examples of ‘normalisation’ of UI by health professionals*

1. Overall, if you had to summarise the impact that these toileting issues have on **your** life, how would you rate this? Please could you point to the one of the faces on the card that best reflects the impact that toileting issues have on your life, where the green face represent no impact, the yellow face represents some impact and the red face, a great deal of impact (supporter).

**SHOWCARD A**

**SECTION: Nocturia**

1. Bladder control, urinary incontinence or having to get up at night to urinate during the night can be a problem for some people. Thinking about how [name of PLWD] has been recently, how often do they pass urine during the night from going to bed to sleep until they get up in the morning?
2. Having to get up at night to urinate can impact on other areas of your life. I wonder if you could describe whether [name of PLWD] having to get up at night to urinate impacts on **your** life? (supporter)

*Conversational Probes*

- *Made it difficult to concentrate the next day*
- *Feel generally low in energy*
- *Difficult to sleep at night, disturbed sleep or getting back to sleep, required to nap the next day*
- *Less productive the next day*
- *Participate less in the activities you enjoy*

1. Overall, if you had to summarise the impact that nocturia had on your life, how would you rate this? Please could you point to the one of the faces on the card that best reflects the impact that toileting issues have on your life, where the green face represent no impact, the yellow face represents some impact and the red face, a great deal of impact (supporter).

**SHOWCARD A**.

**SECTION: UI**

1. Urinary incontinence means a person leaks urine by accident (pees their pants or wets the bed: use participant’s terminology). Does [name of PLWD] ever leak urine?

***Note: If PLWD experiences UI continue with topics below.***

***If PLWD has experienced nocturia that disturbs sleep of supporter skip to 19.***

***If neither nocturia or UI skip to 23.***

1. When does this urine leak?

*Conversation probes*

- *Before can get to toilet*
- *When cough or sneeze*
- *When asleep/at night/bed wetting*
- *Physically active/exercising*
- *Finished dressing and are dressed*
- *No obvious reasons*

1. Do problems with leaking urine interfere with your everyday life (supporter)?

*Conversational probes*

- *Whether they feel embarrassed taking PLWD out or dealing with intimate tasks*
- *Whether they try to make sure they know where the toilets are when they take [name] out*
- *Whether they make plans taking UI into account*
- *Whether UI cause them to stay home more often than they would like*
- *Extra laundry, house cleaning, financial outlay*

1. Overall, if you had to summarise the impact that leaking urine has on your life, how would you rate this? Please could you point to the one of the faces on the card that best reflects the impact that leaking urine has on your life, where the green face represents no impact, the yellow face represents some impact and the red face, a great deal of impact (supporter).

**SHOWCARD A**

**SECTION: Strategies for UI & Nocturia**

***Note: if PLWD experiences both UI and nocturia, probe about strategies for both in topic areas 19-22.***

1. What helps you to deal with [UI and/or nocturia]? How do you manage?

*Conversational probes for*

- *Types of strategies for addressing these problems e.g. incontinence pads, pull-ups, male continence sheath, absorbent bed pad, waterproof mattress protector, catheter, timing of drinking fluids/restricting fluid intake,*
- *For continence products: whether they are provided free or whether they have to purchase them. If they are free, do they have to top up with purchases explore whether provision is inadequate or inappropriate (see below)*
- *Toileting programmes, pelvic floor exercises, bladder retraining*
- *Are there different strategies adopted by PLWD and supporter*
- *Ability to clean up after an episode of urinary incontinence or leakage*

1. Are the strategies you use for [UI and/or nocturia] useful?

*Conversational probes*

- *Preferences about different strategies do they differ between PLWD and supporter*
- *Feelings about different strategies e.g. How do you feel about managing in that way?*
- *Perceived effectiveness and whether this differs between PLWD and supporter.*
- *Are the PLWD and supporter able to use these strategies when they leave the home e.g. visiting friends, shopping, eating out, leisure activities.*

1. **FOR THOSE USING INCONTINENCE AIDS ONLY:** Are there any particular challenges that you face when using incontinence products?

*Conversational probes*

- *Appeal/resistance: if continence aids are useful and acceptable to the PLWD and caregiver, whether they would prefer something else (e.g. pull ups instead of pads)*
- *Product performance: Leakage from products (e.g. pad or continence sheets), device movement/loss of adhesion when walking*
- *Accurate, effective application (by self or carers)*
- *Difficult anatomy, thin skin, excessive tissue/fat, creases and skin folds*
- *Options for sustainable or washable/reusable products (e.g. pads or knickers)*
- *Odour*
- *Skin health e.g. friction, pressure sores, dermatitis/rash, removal of device without skin trauma*
- *Reduced or no potential for toileting*
- *Taking spare products and/or disposal when out*
- *Method and cost of disposal at home*

1. Some people discuss these sorts of problems with their family doctor or with a district or continence nurse. Have you sought advice or help on this from any professional?

*Conversational probes:*

- *With whom (GP community nurse, occupational therapist, physio, mental health nurse)*
- *What support/information was provided*
- *Was this enough support/information: perceptions of efficacy, usefulness, nuisance from the perspectives of PLWD and supporter, any unmet needs*
- *Views on experience of discussing or receiving help from health professionals*
- *Any examples of ‘normalisation’ of UI by health professionals*

**SECTION: Bowels**

1. Some people experience problems with their bowels, getting constipated and then sometimes very loose motions, feacal leakage or uncontrollable wind, does [name of PLWD] ever have those sorts of problems?

*If yes, prompt what type of problem and SHOWCARD B and ask participant to indicate bowel movements*

***SHOWCARD B***

***Note: If participant experiences FI or constipation continue with topics below, otherwise skip to closing section topic 31***

1. Thinking about how [name of PLWD] has been recently, how often does this happen?

*Conversational probe for each type of bowel problem mentioned*

*FOR FI Conversation probes around when this happens*

- *Small amounts leaking all the time*
- *When asleep/at night in bed*
- *When passive e.g. Just sitting*
- *Before they can get to the toilet*
- *At particular points (e.g. after meals)*

1. Do these problems with [leakage of faeces or constipation] interfere with your everyday life (supporter)?

*Conversational probes*

- *Whether they feel embarrassed taking PLWD out or dealing with intimate tasks*
- *Whether they try to make sure they know where the toilets are*
- *Whether they make plans taking FI/constipation into account*
- *Whether FI/constipation causes them to stay home more often than they would like*

*For night time events, if mentioned:*

- *Made it difficult to concentrate the next day*
- *Feel generally low in energy*
- *Difficult to sleep at night, disturbed sleep or getting back to sleep, required to nap the next day*
- *Less productive the next day*
- *Participate less in the activities you enjoy*
- *Careful about how much you drink or when you drink*
- *Concern about disturbing other in the house (e.g. their sleep)*

1. What helps you to deal with [constipation or faecal incontinence]? How do you manage?

***If both then prompt around types of strategies used for each in topic areas 27-29***

*Conversational probes*

- *Types of strategies for addressing these problems e.g. pads, incontinence sheets, colostomy.*
- *For continence products: whether they are provided free or whether they have to purchase them*
- *Are there different strategies adopted by PLWD and supporter*
- *Ability to clean up after an episode of faecal incontinence or leakage.*

1. Are the strategies you use useful?

*Conversational probes*

- *Preferences about different strategies do they differ between PLWD and supporter*
- *Feelings about different strategies e.g. How do you feel about managing in that way?*
- *Perceived effectiveness and whether this differs between PLWD and supporter.*
- *Are the PLWD and supporter able to use these strategies when they leave the home e.g. visiting friends, shopping, eating out, leisure activities.*

1. FOR THOSE USING INCONTINENCE AIDS ONLY: Are there any particular challenges that you face when using incontinence products for faecal incontinence?

***Note: If these topics have been covered in relation to UI, only ask if there are any particular challenges associated with FI that are different.***

*Conversational probes*

- *Appeal/resistance: if continence aids are useful and acceptable to the PLWD and caregiver, whether they would prefer something else (e.g. pull ups instead of pads)*
- *Product performance: Leakage from products, device movement/loss of adhesion when walking*
- *Accurate, effective application (by self or carers)*
- *Difficult anatomy, thin skin, excessive tissue/fat, creases and skin folds*
- *Options for sustainable or washable/reusable products (e.g. pads or knickers)*
- *Odour*
- *Skin health e.g. friction, pressure sores, dermatitis/rash, removal of device without skin trauma*
- *Reduced or no potential for toileting*
- *Taking spare products and/or disposal when out*
- *Method and cost of disposal at home*

1. Some people discuss these sorts of problems with their family doctor or with a district or continence nurse. Have you sought advice or help on this from any professional?

*Conversational probes:*

- *With whom (GP community nurse, occupational therapist, physio, mental health nurse)*
- *What support/information was provided*
- *Was this enough support/information: perceptions of efficacy, usefulness, nuisance from the perspectives of PLWD and supporter, any unmet needs*
- *Views on experience of discussing or receiving help from health professionals*
- *Any examples of ‘normalisation’ of UI by health professionals*

1. Overall, if you had to summarise the impact that [faecal incontinence, constipation] has on your life, how would you rate this? Please could you point to the one of the faces on the card that best reflects the impact that [faecal incontinence, constipation] has on your life, where the green face represents no impact, the yellow face represents some impact and the red face, a great deal of impact (supporter).

**SHOWCARD A**

***Note, ask this twice if PLWD experiences both faecal incontinence and constipation, once for each.***

**SECTION: Spirituality, QOL & Close**

1. Is there anything you would like to tell me about your spirituality, faith or religion that helps or hinders you in dealing with your memory problems and continence issues?

*Conversational probes*

- *Whether they find comfort in religion or spiritual beliefs*
- *Whether illness of [name of PLWD] has strengthened religion or spiritual beliefs.*

1. We’ve talked about lots of things today. Thinking about all of these things in the last week, how would you rate your quality of life overall? Please could you point to the one of the faces on the card that best reflects how you feel, where the faces represent good, fair and poor quality of life.

**SHOWCARD C**

Approved by Southern Health and Disability Ethics Committee on 28/04/2022 for three years. Reference Number 11658
