# Supplementary material for: Improving continence management for people with dementia in the community in Aotearoa, New Zealand: Protocol for a mixed methods study
Source: PLoS One. 2023 Jul 18;18(7):e0288613. doi: 10.1371/journal.pone.0288613 (PMC10353819; doi:10.1371/journal.pone.0288613)
Supplement: S4 Appendix — (DOCX) [file pone.0288613.s004.docx]

**S4 APPENDIX: TOPIC GUIDE PLWD LONGITUDINAL INTERVIEW**

**INTRODUCTORY SECTION**

**Note to interviewers –** all questions are **about the PLWD**. Those that can only be answered by the PLWD (i.e. those that are subjective) are marked with *. Remember there is an option to split interview between two visits if needed.

*To PLWD –*

1. Can you tell me a little bit about yourself - what’s been happening since I last visited you [in month]? (*whatever the participant would like to tell you)*

***DEMOGRAPHIC SURVEY ITEMS ASKED HERE (Wave 2-4 version)***

1. Last time you told me that you were:

[Previous wave summary about ADLs/aids and adaptations or tech used to support ADLs]

Has anything changed since our last interview? How are you managing around the home at the present time?

*Conversational probes (this is to get a general idea about how the PLWD manages, not a huge amount of detail is required)*

- *Activities of daily living such as eating and drinking, dental care, washing, bathing, dressing, physical activity and exercising, personal grooming, home maintenance, laundry, other household chores.*
- *Aids, adaptations or technology used to support activities of daily living*

1. Last time you told me that you were:

[Previous wave summary about doing things in the community]

Has anything changed since our last interview? How are you managing to do the things in the community at the present time?

*Conversational probes (this is to get a general idea about how the PLWD manages, not a huge amount of detail is required)*

- *Physical activity and exercise outside of the home*
- *Telling people about memory problems*
- *Eating and drinking out*
- *Maintaining relationships with people in the community*
- *Participation in religious activity/church/spiritual groups*
- *Travelling and transport*
- *Aids or strategies that are used to support these things*

4.* Last time you told me that your sources of hope, strength, comfort and peace were:

[Previous wave summary about sources of hope, strength, comfort and peace or that they had no religious or spiritual beliefs]

Has anything changed since our last interview with regard to your sources of hope, strength, comfort and peace? For example, a religious or spiritual beliefs acts as a source of comfort and strength for some people in dealing with life's ups and downs.

*Conversational probes*

- *Part of an* ***organized religion*** *if so what is it?*
- ***Personal*** *spirituality/ practices independent of organized religion*
- *How important is spirituality, faith or religion is to the person*

1. Today, I’m going to be talking a lot about getting to and using the toilet, the accidental loss or leaking of urine (e.g. peeing pants, wetting the bed) the accidental loss or leaking faeces, and constipation. Last time you told me you preferred to call….

[Previous wave preferred terminology]

Are you still happy for me to use these terms?

***M-ACE QUESTIONS ASKED HERE (Version B @ Wave 2; Version C @ Wave 3; Version A @ Wave 4)***

**SECTION: Toileting**

1. Some people sometimes experience problems with going to the toilet (e.g. finding the toilet in time, managing clothing). Last time you told me:

[Previous wave summary about toileting]

Has anything changed since our last interview?

*Conversational probes for types of problems*

- *Can’t find the toilet*
- *Finds the toilet but can’t use it*
- *Hides evidence of accidents*
- *Won’t sit on the toilet*
- *Uses inappropriate receptacle/place*
- *Can’t manage clothing*
- *Faecal smearing*
- *Not recognizing the urge or desire to act on it*

*Conversational probe about frequency (e.g. how often does that happen?)*

***If there are toileting problems ask topics 7-12a/b if not skip to 13***

7.* Do these problems with toileting interfere with your everyday life (PLWD)?

*Conversational probes*

- *Whether using the toilet is always on their mind*
- *Whether they feel embarrassed*
- *Whether they try to make sure they know where the toilets are (e.g. in the community or when visiting others)*
- *Whether they make plans taking toileting problems into account*
- *Whether toileting problems cause them to stay home more often than they would like*

1. What helps you to deal with that? How do you manage?

*Conversational probes*

- *Types of strategies for addressing these problems*
- *Are there different strategies adopted by PLWD and supporter*

1. Are the strategies you use useful?

*Conversational probes*

- *Preferences about different strategies do they differ between PLWD and supporter*
- *Feelings about different strategies e.g. How do you feel about managing in that way?*
- *Perceived effectiveness and whether this differs between PLWD and supporter.*

1. Some people discuss these sorts of problems with their family doctor or with a district or continence nurse. Have you sought advice or help on this from any professional?

*Conversational probes:*

- *With whom (GP community nurse, occupational therapist, physio, mental health nurse)*
- *What support/information was provided*
- *Was this enough support/information: perceptions of efficacy, usefulness, nuisance from the perspectives of PLWD and supporter, any unmet needs*
- *Any examples of ‘normalisation’ of UI by health professionals*

1. Have you sought advice or received any help or information about getting to and using the toilet from any other source?

*Conversational probes:*

- *What information or help was being sought*
- *Where did they seek information or help (people, places, organisations/support groups, websites)*
- *What support/information was provided*
- *Did they get the answers they needed, and if so from whom/where (people, places, organisations/support groups, websites)*
- *Is this perceived to be a trusted source/accurate information*
- *Views on experience of discussing or receiving help or information from these sources*

12a. * Overall, if you had to summarise the impact that these toileting issues have on your life, how would you rate this? Please could you point to the one of the faces on the card that best reflects the impact that toileting issues have on your life, where the green face represent no impact, the yellow face represents some impact and the red face, a great deal of impact (PLWD).

**SHOWCARD A**

12b. **ONLY ASK IF THE SUPPORTER IS PRESENT AND HAS NOT REQUESTED A SEPARATE INTERVIEW:** Overall, if you had to summarise the impact that these toileting issues have on your life, how would you rate this? Please could you point to the one of the faces on the card that best reflects the impact that toileting issues have on your life, where the green face represent no impact, the yellow face represents some impact and the red face, a great deal of impact (supporter).

**SHOWCARD A**

**SECTION: Nocturia**

1. Bladder control, urinary incontinence or having to get up at night to urinate during the night can be a problem for some people. Last time you told me:

[Previous wave summary about nocturia]

Has anything changed since our last interview? Thinking about how you have been recently, how often do you pass urine during the night from going to bed to sleep until you get up in the morning?

14. * Having to get up at night to urinate can impact on other areas of your life. I wonder if you could describe whether having to get up at night to urinate has had any impact on you recently (PLWD)?

*Conversational Probes*

- *Made it difficult to concentrate the next day*
- *Feel generally low in energy*
- *Difficult to sleep at night, disturbed sleep or getting back to sleep, required to nap the next day*
- *Less productive the next day*
- *Participate less in the activities you enjoy*
- *Careful about how much you drink or when you drink*
- *Worried about disturbing other in the house (e.g. their sleep)*
- *Falling at night*
- *Ask how often each happens (e.g. frequently, occasionally)*

15a. * Overall, if you had to summarise the impact that nocturia had on your life, how would you rate this? Please could you point to the one of the faces on the card that best reflects the impact that toileting issues have on your life, where the green face represent no impact, the yellow face represents some impact and the red face, a great deal of impact (PLWD).

**SHOWCARD A**.

15b. **ONLY ASK IF THE SUPPORTER IS PRESENT AND HAS NOT REQUESTED A SEPARATE INTERVIEW:** Overall, if you had to summarise the impact that nocturia had on your life, how would you rate this? Please could you point to the one of the faces on the card that best reflects the impact that toileting issues have on your life, where the green face represent no impact, the yellow face represents some impact and the red face, a great deal of impact (supporter).

**SHOWCARD A**.

**SECTION: UI**

1. Urinary incontinence means a person leaks urine by accident (pees their pants or wets the bed: use participant’s terminology).  Last time you told me:

[Previous wave summary about UI]

Has anything changed since our last interview? Do you ever leak urine?

***Note: If participant experiences UI continue with topics below.***

***If no UI, but participant has experienced nocturia that disturbs sleep skip to 20.***

***If neither nocturia nor UI skip to 28***

1. When does this urine leak?

*Conversation probes*

- *Before can get to toilet*
- *When cough or sneeze*
- *When asleep/at night/bed wetting*
- *Physically active/exercising*
- *Finished dressing and are dressed*
- *No obvious reasons*

18*. Do problems with leaking urine interfere with your everyday life (PLWD)?

*Conversational probes*

- *Whether using the toilet is always on their mind*
- *Whether they feel embarrassed*
- *Whether they try to make sure they know where the toilets are*
- *Whether they make plans taking UI into account*
- *Whether UI cause them to stay home more often than they would like*

19a.* Overall, if you had to summarise the impact that leaking urine has on your life, how would you rate this? Please could you point to the one of the faces on the card that best reflects the impact that leaking urine has on your life, where the green face represents no impact, the yellow face represents some impact and the red face, a great deal of impact (PLWD).

**SHOWCARD A**

19b. **ONLY ASK IF THE SUPPORTER IS PRESENT AND HAS NOT REQUESTED A SEPARATE INTERVIEW:** Overall, if you had to summarise the impact that leaking urine has on your life, how would you rate this? Please could you point to the one of the faces on the card that best reflects the impact that leaking urine has on your life, where the green face represents no impact, the yellow face represents some impact and the red face, a great deal of impact (supporter). **SHOWCARD A**

**SECTION: Strategies for UI & Nocturia**

***Note: if person experiences both UI and nocturia, probe about strategies for both in topic areas 20-27.***

1. What helps you to deal with [UI and/or nocturia]? How do you manage?

*Conversational probes for*

- *Types of strategies for addressing these problems e.g. incontinence pads, pull-ups, male continence sheath, absorbent bed pad, waterproof mattress protector, catheter,*
- *For continence products: whether they are provided free or whether they have to purchase them. If they are free, do they have to top up with purchases explore whether provision is inadequate or inappropriate (see below)*
- *Toileting programmes, pelvic floor exercises, bladder retraining*
- *Are there different strategies adopted by PLWD and supporter*
- *Ability to clean up after an episode of urinary incontinence or leakage*

1. Have you maintained or changed any particular drinking habits to help manage [UI and/or nocturia]? Note this includes water.

*Conversational probes for*

- *Timing of drinking fluids*
- *Restricting or increasing fluid intake*
- *Restricting or increasing particular types of drinks (e.g. alcohol, coffee)*
- *Including different types of drinks (e.g. kombucha, cranberry juice, barley water)*

1. Have you maintained or changed any particular eating habits to help manage [UI and/or nocturia]?

*Conversational probes for*

- *Timing of eating*
- *Restricting or increasing particular types of food*
- *Including different types of food*

1. Do you take any medication, herbal, natural remedies, or use complementary therapies or rongoa that you think influence [UI and/or nocturia]?

*Conversational probes for*

- *Use of prescribed, over the counter medication, therapies or rongoa that increases/decreases urine output*
- *Self-management of medication or Rongoa for particular occasions (e.g. not taking a diuretic before going shopping)*

1. Are any of the strategies you have mentioned that you use for [UI and/or nocturia] useful?

*Conversational probes*

- *Preferences about different strategies do they differ between PLWD and supporter*
- *Feelings about different strategies e.g. How do you feel about managing in that way?*
- *Perceived effectiveness and whether this differs between PLWD and supporter.*
- *Are the PLWD and supporter able to use these strategies when they leave the home e.g. visiting friends, shopping, eating out, leisure activities.*

1. FOR THOSE USING INCONTINENCE AIDS ONLY: Are there any particular challenges that you face when using incontinence products?

*Conversational probes*

- *Appeal/resistance: if continence aids are useful and acceptable to the PLWD and caregiver, whether they would prefer something else (e.g. pull ups instead of pads)*
- *Product performance: Leakage from products (e.g. pad or continence sheets), device movement/loss of adhesion when walking*
- *Accurate, effective application (by self or carers)*
- *Difficult anatomy, thin skin, excessive tissue/fat, creases and skin folds*
- *Options for sustainable or washable/reusable products (e.g. pads or knickers)*
- *Odour*
- *Skin health e.g. friction, pressure sores, dermatitis/rash, removal of device without skin trauma*
- *Reduced or no potential for toileting*
- *Taking spare products and/or disposal when out*
- *Method and cost of disposal at home*

1. Some people discuss these sorts of problems with their family doctor or with a district or continence nurse. Have you sought advice or help on this from any professional?

*Conversational probes:*

- *With whom (GP community nurse, occupational therapist, physio, mental health nurse)*
- *What support/information was provided*
- *Was this enough support/information: perceptions of efficacy, usefulness, nuisance from the perspectives of PLWD and supporter, any unmet needs*
- *Views on experience of discussing or receiving help from health professionals*
- *Any examples of ‘normalisation’ of UI by health professionals*

1. Have you sought advice or received any help or information about [nocturia/UI] from any other source?

*Conversational probes:*

- *What information or help was being sought*
- *Where did they seek information or help (people, places, organisations/support groups, websites)*
- *What support/information was provided*
- *Did they get the answers they needed, and if so from whom/where (people, places, organisations/support groups, websites)*
- *Is this perceived to be a trusted source/accurate information*
- *Views on experience of discussing or receiving help or information from these sources*

**SECTION: Bowels**

1. Some people experience problems with their bowels, getting constipated and then sometimes very loose motions, feacal leakage or uncontrollable wind. Last time you told me:

[Previous wave summary about bowels]

Has anything changed since the last interview? Do you ever have those sorts of problems?

*If yes, prompt what type of problem and SHOWCARD B and ask participant to indicate bowel movements*

***SHOWCARD B***

***Note: If participant experiences faecal incontinence or constipation continue with topics below, otherwise skip to services provision topic 40.***

1. Thinking about how you have been recently, how often does this happen?

*Conversational probe for each type of bowel problem mentioned*

*FOR FI Conversation probes around when this happens*

- *Small amounts leaking all the time*
- *When asleep/at night in bed*
- *When passive e.g. Just sitting*
- *Before they can get to the toilet*
- *At particular points (e.g. after meals)*

30.* Do these problems with [leakage of faeces or constipation] interfere with your everyday life (PLWD)?

*Conversational probes*

- *Whether using the toilet is always on their mind*
- *Whether they feel embarrassed*
- *Whether they try to make sure they know where the toilets are*
- *Whether they make plans taking FI/constipation into account*
- *Whether FI/constipation causes them to stay home more often than they would like*

*For night time events, if mentioned:*

- *Made it difficult to concentrate the next day*
- *Feel generally low in energy*
- *Difficult to sleep at night, disturbed sleep or getting back to sleep, required to nap the next day*
- *Less productive the next day*
- *Participate less in the activities you enjoy*
- *Careful about how much you drink or when you drink*
- *Concern about disturbing other in the house (e.g. their sleep)*

1. What helps you to deal with [constipation or faecal incontinence]? How do you manage?

***If both then prompt around types of strategies used for each in topic areas 32-39.***

*Conversational probes*

- *Types of strategies for addressing these problems e.g. pads, incontinence sheets, colostomy.*
- *For continence products: whether they are provided free or whether they have to purchase them*
- *Are there different strategies adopted by PLWD and supporter*
- *Ability to clean up after an episode of faecal incontinence or leakage.*

1. Have you maintained or changed any particular drinking habits to help manage [constipation or faecal incontinence]? Note includes water.

*Conversational probes for*

- *Timing of drinking fluids e.g. hot drink in the morning*
- *Restricting or increasing fluid intake*
- *Restricting or increasing particular types of drinks (e.g. alcohol, coffee)*
- *Including different types of drinks (e.g. kombucha, kiwi crush, prune juice)*

1. Have you maintained or changed any particular eating habits to help manage [constipation or faecal incontinence]?

*Conversational probes for*

- *Timing of eating*
- *Restricting or increasing particular types of food*
- *Including different types of food*

1. Do you take any medication, herbal, natural remedies, or use complementary therapies or rongoa that you think influence [constipation or faecal incontinence]?

*Conversational probes for*

- *Use of prescribed, over the counter medication, therapies or rongoa (e.g. laxative or anti-diarrhoea)*
- *Self-management of medication or rongoa for particular occasions (e.g. not taking a laxative before going out)*

1. Are any of the strategies you have mentioned that you use for [faecal incontinence, constipation] useful?

*Conversational probes*

- *Preferences about different strategies do they differ between PLWD and supporter*
- *Feelings about different strategies e.g. How do you feel about managing in that way?*
- *Perceived effectiveness and whether this differs between PLWD and supporter.*
- *Are the PLWD and supporter able to use these strategies when they leave the home e.g. visiting friends, shopping, eating out, leisure activities.*

36. FOR THOSE USING INCONTINENCE AIDS ONLY: Are there any particular challenges that you face when using incontinence products for faecal incontinence?

***Note: If these topics have been covered in relation to UI, only ask if there are any particular challenges associated with FI that are different.***

*Conversational probes*

- *Appeal/resistance: if continence aids are useful and acceptable to the PLWD and caregiver, whether they would prefer something else (e.g. pull ups instead of pads)*
- *Product performance: Leakage from products, device movement/loss of adhesion when walking*
- *Accurate, effective application (by self or carers)*
- *Difficult anatomy, thin skin, excessive tissue/fat, creases and skin folds*
- *Options for sustainable or washable/reusable products (e.g. pads or knickers)*
- *Odour*
- *Skin health e.g. friction, pressure sores, dermatitis/rash, removal of device without skin trauma*
- *Reduced or no potential for toileting*
- *Taking spare products and/or disposal when out*
- *Method and cost of disposal at home*

1. Some people discuss these sorts of problems with their family doctor or with a district or continence nurse. Have you sought advice or help on this from any professional?

*Conversational probes:*

- *With whom (GP community nurse, occupational therapist, physio, mental health nurse)*
- *What support/information was provided*
- *Was this enough support/information: perceptions of efficacy, usefulness, nuisance from the perspectives of PLWD and supporter, any unmet needs*
- *Views on experience of discussing or receiving help from health professionals*
- *Any examples of ‘normalisation’ of UI by health professional*

1. Have you sought advice or received any help or information about [faecal incontinence, constipation] from any other source?

*Conversational probes:*

- *What information or help was being sought*
- *Where did they seek information or help (people, places, organisations/support groups, websites)*
- *What support/information was provided*
- *Did they get the answers they needed, and if so from whom/where (people, places, organisations/support groups, websites)*
- *Is this perceived to be a trusted source/accurate information*
- *Views on experience of discussing or receiving help or information from these sources*

39a.* Overall, if you had to summarise the impact that [faecal incontinence, constipation] has on your life, how would you rate this? Please could you point to the one of the faces on the card that best reflects the impact that [faecal incontinence, constipation] has on your life, where the green face represents no impact, the yellow face represents some impact and the red face, a great deal of impact (PLWD).

**SHOWCARD A**

***Note, ask this twice if person experiences both faecal incontinence and constipation, once for each.***

39b. **ONLY ASK IF THE SUPPORTER IS PRESENT AND HAS NOT REQUESTED A SEPARATE INTERVIEW:** Overall, if you had to summarise the impact that [faecal incontinence, constipation] has on your life, how would you rate this? Please could you point to the one of the faces on the card that best reflects the impact that [faecal incontinence, constipation] has on your life, where the green face represents no impact, the yellow face represents some impact and the red face, a great deal of impact (PLWD).

**SHOWCARD A**

***Note, ask this twice if PLWD experiences both faecal incontinence and constipation, once for each.***

**SECTION: Service Provision**

We’ve talked today about your experiences with the health services and health professionals in supporting you to promote continence and manage incontinence. I would like to explore this in a little more detail.

Across your life, have any of your previous experiences with public and private sector services influenced how you feel about accessing the health service or health professional support today?

*Conversational probes:*

*Education*

*Health services*

*Private sector*

Based on your previous experiences of the health sector, can you tell me about a time when you had an experience with a health professional or service that was really great, and describe what made it such a good experience?

*Conversational probes:*

- *Developing relationships and building rapport*
- *Supporting dignity and mana*
- *Providing privacy, respecting personal space and confidentiality*
- *Being treated with care and compassion*
- *Communicating and listening*
- *Information, choices, and decision making (individual and with whānau/care partners)*
- *Access to right types and levels of help/care/support/treatment*
- *Connection between different services/transitions between services*
- *Acknowledges spirituality, religion and cultural practices*
- *Are there different life course experiences noted for PLWD and supporter?*

Based on your previous experiences of the health sector, can you tell me about a time when you had an experience with a health professional or service that was not very good, and described what made it such a bad experience?

*Conversational probes:*

- *Developing relationships and building rapport*
- *Supporting dignity and mana*
- *Providing privacy, respecting personal space and confidentiality*
- *Being treated with care and compassion*
- *Communicating and listening*
- *Information, choices, and decision making (individual and with whānau/care partners)*
- *Access to right types and levels of help/care/support/treatment*
- *Connection between different services/transitions between services*
- *Acknowledges spirituality, religion and cultural practices*
- *Are there different life course experiences noted for PLWD and supporter?*

**SECTION: Spirituality, QOL & Close**

1. Is there anything you would like to tell me about your spirituality, faith or religion that helps or hinders you in dealing with your memory problems and continence issues?

*Conversational probes*

- *Whether they find comfort in religion or spiritual beliefs*
- *Whether illness has strengthened religion or spiritual beliefs.*

1. Do you have any proverb or saying that reflects your continence journey?

45a.* We’ve talked about lots of things today. Thinking about all of these things in the last week, how would you rate your quality of life overall? Please could you point to the one of the faces on the card that best reflects how you feel, where the faces represent good, fair and poor quality of life.

**SHOWCARD C**

45b.  **ONLY ASK IF THE SUPPORTER IS PRESENT AND HAS NOT REQUESTED A SEPARATE INTERVIEW:** We’ve talked about lots of things today. Thinking about all of these things in the last week, how would you rate your quality of life overall? Please could you point to the one of the faces on the card that best reflects how you feel, where the faces represent good, fair and poor quality of life.

**SHOWCARD C**

Approved by Southern Health and Disability Ethics Committee on 28/4/2022 for three years. Reference Number 11658
