# Supplementary material for: Improving continence management for people with dementia in the community in Aotearoa, New Zealand: Protocol for a mixed methods study
Source: PLoS One. 2023 Jul 18;18(7):e0288613. doi: 10.1371/journal.pone.0288613 (PMC10353819; doi:10.1371/journal.pone.0288613)
Supplement: S6 Appendix — (DOCX) [file pone.0288613.s006.docx]

Inclusivity in global research

PLOS’ policy on inclusivity in global research aims to improve transparency in the reporting of research performed outside of researchers’ own country or community and ensures that PLOS publications reporting global research adhere to high standards for research ethics and authorship. Authors of relevant research articles may be asked to complete the questionnaire below, which outlines ethical, cultural, and scientific considerations specific to inclusivity in global research. This questionnaire may be requested when researchers have travelled to a different country to conduct research, if research uses samples collected in another country, research with Indigenous populations or their lands, or if research is on cultural artefacts. Researchers travelling to another country solely to use laboratory equipment will not normally be required to complete the questionnaire. However, the questionnaire can be requested at the journal’s discretion for any submission – if you have been requested to complete this questionnaire by the PLOS journal you submitted to, please do so.

Please complete the questionnaire below and include this as a Supporting Information file with your manuscript. Note that if your paper is accepted for publication, this checklist will be published with your article in the supporting information files. Please ensure that you reference the checklist in the main body of your manuscript. We suggest adding a subsection ‘Inclusivity in global research’ to your Methods section and adding the following sentence: “Additional information regarding the ethical, cultural, and scientific considerations specific to inclusivity in global research is included in the Supporting Information (SX Checklist)”

The questions have been designed to be applicable to a wide range of study types, and there are subsections for both human subjects research and non-human subjects research. If any of the questions are not relevant to your research please mark them as “N/A” as appropriate.

**Ethical considerations, permits and authorship**

*This section is applicable to all research types.*

Provide details as to who granted permissions and/or consent for the study to take place in the Methods section of your manuscript. This should include the names of **all** ethics boards, governmental organizations, community leaders or other bodies that provided approval for the study. If individuals provided approval refer to these people by their role or title but do not list their name(s).

Ethical approval for Phase 1 was obtained from the Auckland Health Research Ethics Committee (reference AH23238) on the 1 September 2021.

Ethical approval for Phase 2 was obtained from the Auckland Health Research Ethics Committee (reference AH23747) on the 10 October 2021. In Phase 2, health professionals will be provided with a participant information sheet and will be asked to provide written consent to participate in focus groups.

Ethics approval for Phase 3 was obtained from Southern Health and Disability Ethics Committee (reference 11658) on the 28 April 2022. After first contact with the research team (via email or phone as indicated on the advert), potential participants will be sent a copy of the Participant Information Sheet (PIS) by post or email. Separate PIS will be provided for caregivers and PLWD, and for cross-sectional and longitudinal interviews. The research team will follow-up after two weeks and make an appointment for interview if the potential participant wishes to take part in the study. At this time, participants will be asked if they have any more questions about the study that they would like answered. Participants will be asked to give written informed consent on the day of the interview, prior to the interview commencing. Judgments concerning capacity to provide informed consent will be made by trained researchers. The researchers will ask participants to describe in their own words what the study is about, and what they are being asked to do. Consent will be on-going throughout the study and participants will be asked to consent (with the relevant judgements of capacity made) at every data collection point.

Ethical approval for Phase 4 will be sought towards the end of Year 2 (e.g., September 2023). Participants in face-to-face focus groups will be asked to provide written consent, and participants completing written or online surveys will be asked to indicate consent by agreeing to a privacy and consent section before submitting the survey.

If there were any deviations from the study protocol after approval was obtained please provide details of these changes in the Methods section of your manuscript.
Did this study involve local collaborators that are residents of the country where the research was conducted or members of the community studied? If you do not have any authors from said communities, please provide an explanation for this below.

The PI and Co-Is live and work in New Zealand. Indigenous people (Māori) are Co-Is and members of the research team: Sharon Awatere (SA), Tess Moeke-Maxwell (TMM) and Arapera Riki (AR) are Māori researchers. SA and AR live and work in the regions where Māori participants are being recruited from.

Reported on page number: N/A

Everyone listed as an author should meet PLOS’ criteria for authorship and all individuals who meet these criteria should be included in the author byline, rather than the acknowledgements. Authorship criteria is based on the International Committee of Medical Journal Editors (ICMJE) Uniform Requirements for Manuscripts Submitted to Biomedical Journals - for further information please see here: <https://journals.plos.org/plosone/s/authorship>.

**Human subjects research (e.g. health research, medical research, cross-cultural psychology)**

Did you obtain written informed consent from a representative of the local community or region before the research took place? How did you establish who speaks for the community? Details of written informed consent obtained from study participants should be reported separately in the Methods section of your manuscript.

The ethical approval in New Zealand via AHREC and HDEC required the applications to state relevance to Māori. Applications are reviewed accordingly by Māori that sit on the research ethics committees.

Informed consent is taken directly from participants and is reported in the methods section of our protocol.

How did members of the local community provide input on the aims of the research investigation, its methodology, and its anticipated outcome(s)?

This research proposal was co-produced by Māori researchers and partners including TMM, and Mahitahi Hauora PHE (Davis, Nurse Director). The Māori Health Population Health & Strategy Team of Northland DHB; and Mahitahi Hauora PHE have confirmed the research is needed to understand the experiences and support needs of Māori caregivers and whānau promoting continence and managing incontinence (addressed in Phase 3).

The principles of co-production and partnership that underpin this proposal (Mahi tahi: collaboration and equitable involvement in research; Kotahitanga: solidarity and capacity-building; Rangatiratanga: empowerment and action for systems change; and Kaitiakitanga: sustainability) are aligned to Te Tiriti o Waitangi.

Ethical approval has been sought from the Faculty of Health and Medical Research Ethics Committee, University of Auckland and Health and Disability Ethics Committee, both whcih include Māori members and review the protocol for relevance to Māori.

The team is building Māori community researcher capability in Māori health research through mentorship by TMM of SA and AR. TMM is also providing cultural guidance in kaupapa Māori and mātauranga kaumātua informed research processes. The use of Kāhui Kaumātua is consistent with kaupapa Māori principles.

The collaborative partnerships established between SA, AR in Hawkes Bay demonstrates a strong and sustained relationship of mutual respect that benefit Māori kaumātua and whānau manaaki.

In phases 2-4 recruitment of Māori service providers and Māori kaumatua, and whānau manaaki will be informed by a pōwhiri model of engagement to ensure the rangatiratanga (whakapapa, mana, tikanga) of each participant whānau is cared for in a culturally meaningful reciprocal knowledge exchange, based on the principles of whanaungatanga and manaakitanga.

All research with Māori participants will be led by Sharon Awatrere (Māori Research Fellow) and supported by an experienced advisor Moeke-Maxwell (TMM; Ngāi Tai ki Tāmaki Makaurau and Ngāti Porou). A Kāhui Kaumātua (advisory group) will support this study. It will provide cultural leadership and governance over Māori research processes including guidance on interpretation of analysis, oversight of dissemination and decisions over the future use of Māori research data through regular meetings with TMM and Māori community researchers.

When engaging with the local community, how did you ensure that the informed consent documents and other materials could be understood by local stakehlders?

Taking consent and interviews are conducted in English rather than Te Reo Māori and are understandable by the local stakeholders (who use both English and Te Reo Māori). Common Te Reo works have been used in the Māori version of the topic guides, and they have been ordered in a way that fits with Te Whare Tapa Whā.

Will the findings of the research be made available in an understandable format to stakeholders in the community where the study was conducted (e.g. via a presentation, summary report, copies of publications, etc.)? Please provide details of how this will be achieved.

Our research plan has been co-designed with community partners and we have developed an integrated knowledge transfer strategy to maximise the impact of outputs from the research within and beyond the grant lifecycle. Co-production with community-based partners, PLWD and caregivers, whānau and family will result in culturally appropriate real-world application.

To ensure effective two-way communication and routes to translation, we have established a kaumātua ropu advising on the resources and dissemination activities that we should deliver to the Māori community. The kaumātua ropu will provide impartial advice, support and guidance based on the objectives of the research. They review progress of the research and assist in building targeted collaborations with academics, industry, third and public sectors, helping with public engagement and dissemination. They will review and advise on planned dissemination and knowledge transfer activities and identify where added value can be obtained from the activities of the research, including through their own role/organisation.

Inequities in health experiences and outcomes are the result of colonialisation and persistent structural inequities that include inadequate cultural competency and safety within health organizations. Health care providers need to work on communicating information that resonates with the diverse cultural values and contexts of Māori, and developing appropriate outreach mechanisms to connect with whānau manaaki. Phase 2 of our study would establish the extent to which continence policies address the needs of Māori kaumātua living with mate wareware in the community and whānau manaaki. In Phase 4, new continence guidelines will ensure that these needs are met as described under Te Whare Tapa Whā, taking into account taha tinana, taha wairua, taha whānau and taha hinengaro.

In Phase 3, this research will identify Māori whānau-based experiences of, and strategies for promoting continence and managing incontinence for kaumātua living with mate wareware. In order to provide optimal care for kaumātua living with mate wareware, whānau require culturally safe practical support and resources to help promote continence and manage incontinence. Based on experiential evidence, the ‘best’ approaches will be identified, co-developed and rolled out in Phase 4 to support kaumātua living with mate wareware and whānau manaaki to meet their needs relating to toileting and continence challenges. Additionally, the core outcome set (Phase 4) will represent Māori preferences and will ensure that future interventions developed in Aotearoa New Zealand intended to promote continence or manage incontinence will also meet Māori needs and are culturally appropriate.

**Non-human subjects research using specimens/ animals collected as part of the study, or those housed in archival collections. Examples include archaeology, paleontology, botany and zoology.**

Did the permission you obtained from a local authority to perform the study include an agreement on access to outputs and benefit sharing? This may include procedures to enable fair distribution of the benefits and resources arising from the research performed. Please include any details of Prior Informed Consent and Benefit Sharing Agreements obtained. These may be required by field-specific regulations, for example the Convention on Biological Diversity (CBD) and the associated Nagoya Protocol.

If the material used in your study was imported, please A) provide the year it was imported and B) indicate whether permits were obtained to import/export the materials used, C) provide details of any permits obtained. If this information is not available, please indicate this.

If you used archival specimens, please state how the material used in your study was acquired by the institute it is held in and provide details of any permits obtained for the original excavations/ sample collection. If this information is not available, please indicate this.

How was the potential cultural significance of the materials collected in your study to local communities considered in your research design? Were Indigenous peoples and/or local researchers and institutions involved with archaeological excavations / collection of specimens? If so, please provide a description of their involvement.

If your manuscript includes photographs of human remains please indicate whether authors obtained permission from descendants or affiliated cultural communities to do so.
